# How do people living with psychotic disorders access and use information and communication technology: a scoping review

**DOI:** 10.3389/fpsyt.2025.1639348

**Published:** 2025-08-07

**Authors:** Jaclin Vozza, Rebecca Ripco, Sandra Moll, Evelyne Durocher, Rebecca Gewurtz

**Affiliations:** 1 School of Rehabilitation Science, McMaster University, Hamilton, ON, Canada; 2 Schizophrenia Outpatient Clinic, St. Joseph’s Healthcare Hamilton, Hamilton, ON, Canada

**Keywords:** serious mental illness, technology, social connection, community participation, psychotic disorders

## Abstract

**Background:**

Community participation and social connection are important in the recovery process for people living with psychotic disorders. Information and Communication Technology (ICT) can play an important role in recovery by supporting community participation and social connection, but little is known about patterns of use or impact of this use among people living with psychotic disorders. There is a need to synthesize this interdisciplinary literature to establish guidelines for practice.

**Methods:**

We conducted a scoping review to answer the primary question; “What has been written about how people living with psychotic disorders access or use ICT for social connection and community participation?”. Sub-questions include: (1) “What are barriers and facilitators to using ICT for people living with psychotic disorders?” and (2) “What are risks and benefits to using ICT for people living with psychotic disorders?”. We searched six interdisciplinary databases to identify relevant peer-reviewed studies for this scoping review. Two authors independently screened titles and abstracts, and the first author reviewed all full-text articles meeting the inclusion criteria, extracting relevant data pertaining to the research question, with the second author reviewing for consensus. A qualitative content analysis was conducted to capture key trends in existing literature related to the research question.

**Results:**

Nineteen studies were included in this analysis. Findings were categorized into four key areas: 1) differences and similarities in ICT use between participants with psychotic disorders and other populations; 2) moderators of ICT use and access; 3) potential benefits of ICT use and access; and 4) potential risks of ICT.

**Conclusions:**

The results of this review suggest that ICT could be an important and influential tool for participants living with psychotic disorders, despite the existence of significant risks. People living with psychotic disorders are at risk of being left behind the general population in terms of access to technology because of the costs associated with many devices and lack of access to digital literacy education and support for their use; this is an issue of equity and justice. It is essential that future practice and research focus upon how to include this population equitably in this critical occupation through direct intervention.

**Systematic Review Registration:**

https://osf.io/, identifier 10.17605/OSF.IO/YUQXD.

## Background

The number of people diagnosed with mental illness continues to grow and it is one of the primary drivers of disability worldwide (1). In Canada alone, over 5 million people, or 18% of the population, met the criteria for a diagnosis of mental illness in the last 12 months (2). Further, more than a third of people living with mental illness have reported unmet or only partially met mental health care needs (2). Adults living with Serious Mental Illness (SMI) who have persistent mental health difficulties involving psychosis face even more significant challenges. People living with psychotic disorders, such as schizophrenia or schizoaffective disorder, have higher mortality rates than the general population, with their life expectancies being shortened by between 10 and 28.5 years (3, 4). This population can also experience reduced community participation and social connection (5, 6). These factors can negatively impact social, physical, and emotional health, which can impact mental illness (7).

Community participation and social connection have been strongly associated with better health outcomes for people with SMI (8–11). In some recent research among various clinical populations with mental illness, social connection and community participation have been shown to help reduce the risk of heart disease (8, 9), stroke (8, 9), dementia (8, 9), depression (9, 11), and anxiety (9, 10). Social connection and community participation have also been associated with better quality of sleep (9), healthy eating habits and physical activity (9), improved ability to manage stress (9),and better overall health and wellbeing (10). Although there has been some concern that participation in virtual communities might increase social isolation (12), online engagement has been positively correlated with civic engagement such as voting among the general population (13, 14). Further, in a momentary sampling study of 339 participants, social media has been shown to expand online social connections among people with SMI, including family and personal relationships, which are maintained both on and offline (15). The authors of one longitudinal study seeking to assess change in psychological health and wellbeing with Internet use conducted with American household members ages 13 to 101, identified that using the Internet for social connection was associated with a decrease in depressive symptoms (16). Social connection via Internet access is an increasingly important part of many people’s lives with and without SMI and seems to have a positive impact on building social networks (17).

Information and Communication Technology (ICT), which for the purpose of this scoping review will be defined as any device used to connect to the Internet and help people interact in the digital world, has become a means for community participation and social connection. As of 2024, it was estimated that there were 5.35 billion Internet users worldwide, with an expected 7.9 billion Internet users by 2029 (18). People with psychotic disorders, however, may face significant barriers to accessing ICT, such as having low income in conjunction with high costs of treatment, unstable housing, and reduced everyday exposure to use of ICT (19, 20). In addition to these barriers, people with psychotic disorders may face cognitive challenges, paranoia, and reduced motivation that can interfere with learning, access and use of technology (19). Given these cumulative barriers, there is a need to consider access and use of ICT among this population.

Social connection is defined as “the size and diversity of one’s social network and roles, the functions these relationships serve, and their positive or negative qualities” (9). For the purpose of this scoping review, community participation is defined as an individual’s participation in their social network or other meaningful activities within their community. This might include volunteering, employment, and caring for community spaces. Though there is little understanding on how people with psychotic disorders use ICT for social connection and community participation, there is some indication that many people with psychotic disorders are willing to use this method of communication (21, 22). However, this literature is fragmented, making it difficult to discern key barriers and facilitators to access and use of ICT among this population, risks and benefits for social connections, and how to best intervene to improve access and use of ICT for social connection to support mental health recovery. ICT use has the potential to be a meaningful occupation or activity that will open up opportunities for further engagement in the recovery process.

The purpose of this scoping review is to map and analyze the peer-reviewed research about the ways in which people living with psychotic disorders access and use ICT for social connection and community participation. The specific research question addressed by this scoping review is: What has been written about access or use of ICT by people living with psychotic disorders for social connection and community participation?

Sub-questions addressed by this scoping review include the following:

1. What are barriers and facilitators to using ICT for people with psychotic disorders?
2. What are risks and benefits to using ICT for people with psychotic disorders?

## Methods

Following the scoping review methodology developed by Arksey and O’Malley (23) and advanced by Levac and colleagues (24), we created a protocol based on the Joanna Briggs Institute (JBI) scoping review criteria (25). The protocol aligns with the Preferred Reporting Items for Systematic reviews and Meta-Analyses extension for Scoping Reviews (PRISMA-ScR) (26), and is registered with the Open Science Framework (https://doi.org/10.17605/OSF.IO/YUQXD).

### Search strategy

Following consultation with a research librarian with expertise in clinical research, the following six databases were searched on August 7^th^, 2024: CINAHL, Embase, Emcare, MEDLINE, PsycINFO, and Web of Science. The purpose of the searches was to identify relevant peer-reviewed, published studies focused on people living with psychotic disorders and how they access and use ICT for social connection and community participation. The search strategy included three key concepts: psychotic disorders, technology, and social connection/community participation. The search strategy for the terms “SMI” and “ICT” were developed on consultation with a research librarian, drawing on the searches described in a systematic review of digital peer support mental health interventions for people with SMI by Fortuna et al. (27). The search strategy focused on different types of ICT use and did not focus on therapeutic interventions. Searches were also developed for social connection and community participation. Searches were limited to peer-reviewed sources, but were not limited to the English language in order to ensure potentially relevant articles were not missed. As an example, the full search strategy for MEDLINE is presented in **Appendix A**.

### Inclusion and exclusion criteria

We included studies that were peer-reviewed, focused on community-dwelling adults (18+) with diagnoses of a primary psychotic disorder, and reported on how this population uses ICT for social connection and community participation. Studies that did not identify a specific diagnosis were excluded. Studies involving perspectives of healthcare clinicians providing care for the target population were also sought and included. When we identified a systematic review that was relevant to our search, we excluded the individual primary studies to ensure there was not duplication.

Conference abstracts, clinical opinion pieces (e.g., letters to the editor), and all non-peer-reviewed sources were excluded. We did not place limits on study design of peer-reviewed sources; qualitative studies and systematic reviews were also considered; no limits were imposed on geographical location. Studies published since 1992 were included, as this year marks the inception of the smartphone and approximate date wherein the Internet began to be more widely used.

### Study selection

All identified studies were imported into Covidence review software, and duplicates were removed. Screening was independently completed by two reviewers, JV and RR, in two phases. In phase one, titles and abstracts of studies were screened for relevance. In phase two, the full text of relevant studies was accessed, reviewed, and screened for eligibility against the identified inclusion and exclusion criteria. The specific list of reasons for exclusion that was used in phase two of screening included: wrong patient population (e.g., a sample that included less than 50% of individuals with a primary psychotic disorder), wrong outcomes (e.g., not specific to social connection or community participation), wrong study design (e.g., opinion piece), wrong intervention (e.g., piloting a self-management smartphone app), and inability to access full text. Studies that included both individuals with a primary psychotic disorder and other mental illnesses were included if the study population clearly stated that the sample was made up of at least 50% of individuals diagnosed with a primary psychotic disorder. Systematic reviews were included as opposed to the individual primary studies when the systematic reviews answered the research questions outlined by this scoping review and the primary studies did not. Any disagreements between the reviewers during both phases of screening were resolved through discussion and inclusion of a third researcher, RG.

### Data charting

A data extraction template (see **Appendix B**) was developed by the research team in Covidence to extract data relevant to the research question. Data items charted for each study included: authors, year of publication, country of origin, study aims/objectives, study design (quantitative, qualitative, or mixed methods), participants/sample characteristics, information about accessing and using ICT, and any additional considerations. The first author completed data charting for each included study, while the second author reviewed the data charting for accuracy. Any disagreements were resolved through discussion. Consistent with scoping review methodology, quality appraisal of the included studies was not completed.

### Data synthesis

This scoping review used qualitative content analysis methods as outlined by Erlingsson and Brysiewicz (28), Kleinheksel and colleagues (29), and Vaismoradi and colleagues (30). The first author independently reviewed the data from the data extraction tables in Dedoose Version 9.0.17, a cloud-based mixed-method software, to develop and manage codes in an inductive and iterative process. Codes were compared, refined, and organized into overarching categories and subcategories, and discussed and reviewed with the research team to finalize the results.

## Results

### Characteristics of included sources

**Figure 1:**
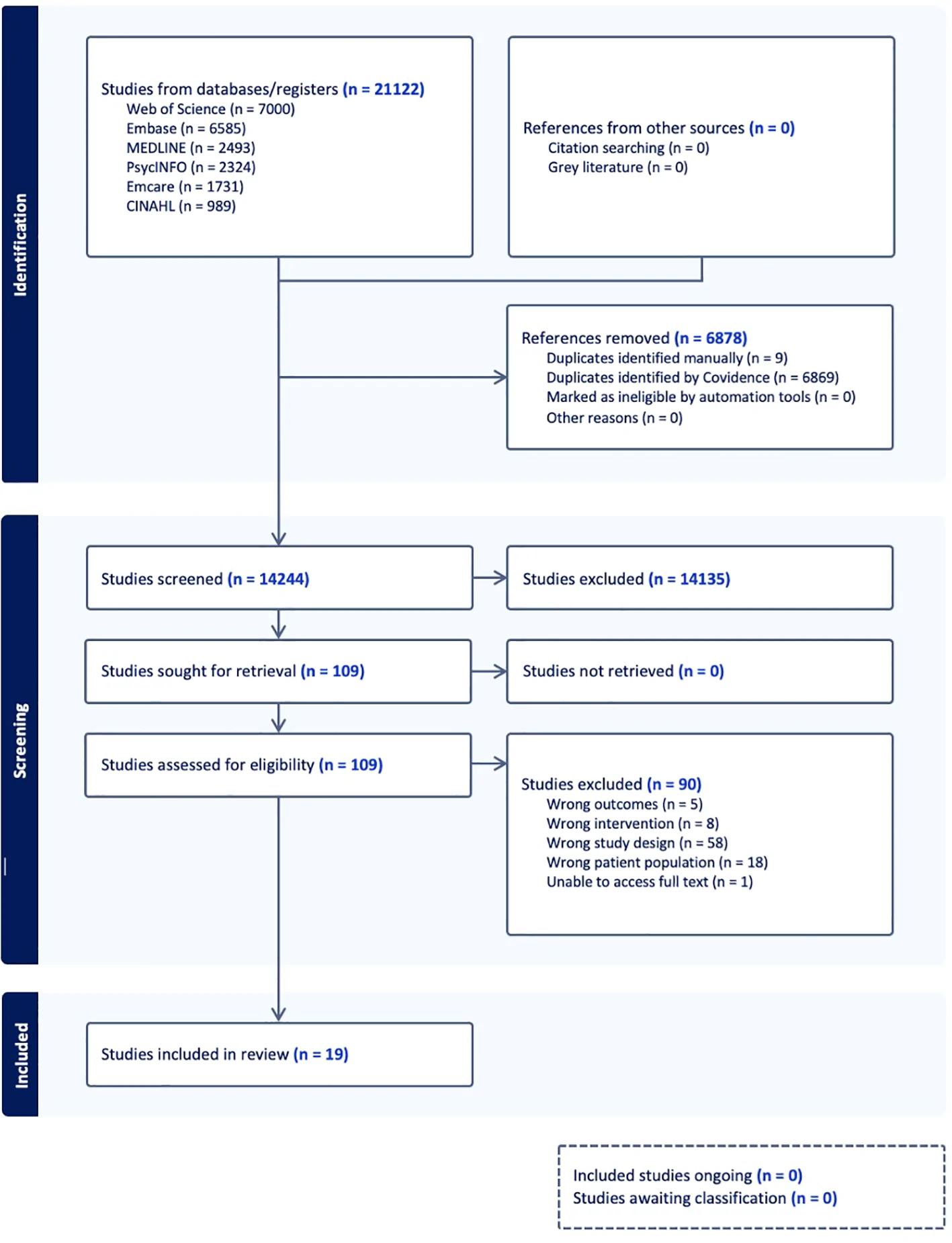
Preferred reporting items for systematic reviews and meta-analyses (PRISMA) flow diagram.

The data base searches yielded a total of 14,244 unique sources after duplicates were removed. A total of 14,135 studies were identified as irrelevant based on screening of their title and abstract; the remaining 109 full texts were assessed for eligibility. At this stage, 90 sources were excluded; exclusion were related to studies being focused on wrong outcomes or outcomes unrelated to the research questions (n = 5), wrong patient population or participant populations that were unrelated to the research questions (n = 18), wrong study design (papers that included grey literature, conference abstracts, opinion pieces, etcetera) (n = 58), inability to access full text (n = 1), and wrong intervention or interventions that were unrelated to the research questions (n = 8). Data charting was completed for the remaining 19 sources that met the eligibility criteria. See **Figure 1** for the Preferred Reporting Items for Systematic reviews and Meta-Analyses (PRISMA) flow diagram.

The 19 included sources included quantitative (n = 15), mixed methods (n = 1), and qualitative (n=3) research studies. Of the quantitative studies (n = 15), 11 were non-experimental studies, 2 were systematic reviews, 1 was a non-randomized experimental study, and 1 was a randomized controlled trial. The countries of origin of the research studies included in this review were the United Stated (n = 6), the United Kingdom (n = 4), Switzerland (n = 1), Canada and the United States in partnership (n = 1), Israel (n = 1), Austria (n = 1), South Africa (n = 1), Turkey (n = 1), Australia (n = 1), Taiwan, (n = 1), and Spain (n = 1). Of all of the included studies, 18 studies included service users’ perspectives, and 1 included both perspectives of service users and providers. For further information about the characteristics of included studies, please see **Appendix C**; please see **Appendix D** for a summary of assigned categories for each included study.

### Trends in ICT use in participants with psychotic disorders

Among the included studies, 6 reported on patterns of ICT use. Some of the studies reported on the type of ICT use by participants with psychotic disorders; others compared amount of use to the general population. The authors of one study from the United Kingdom surveying patients about views on different formats of volunteering suggested that 13.2% of individuals with psychotic disorders do not use ICT, with 57.6% of the participants preferring face-to-face peer support over digital peer support (31). Other authors out of the United Kingdom described the results of a systematic review of how people with psychotic disorders establish social connections and reported that Facebook was the most commonly accessed social media platform by participants with psychotic disorders (32). Spanakis and colleagues, also from the United Kingdom, in their study of 367 participants focused on exploring the use of Internet and digital devices during the pandemic restrictions and its association with physical and mental health changes (33). These authors reported that people with psychotic disorders used ICT for: entertainment or to obtain information (88.9%), online shopping (84.3%), and social connection (84.8%).

In terms of the amount of use, some studies suggested that people with psychotic disorders use ICT less frequently than the general population (33–36). However, the results about the amount of use of ICT by participants living with psychotic disorders is mixed as the authors of four studies suggested that people with psychotic disorders actually use ICT similarly to the general population (32–34, 36).

### Moderators of ICT use and access

Moderators of ICT use and access included mental health- and cognition-related factors, ICT knowledge and interest, age, and other social determinants of health. Fifteen studies specifically reported on moderators of ICT use.

Authors from one study conducted in the United States described the development of a smartphone illness self-management system for people with schizophrenia and reported that clinicians perceived participants with psychotic disorders to have significant difficulty operating a mobile device (37). The results of five additional studies suggest reduced cognitive function secondary to psychosis can limit participants’ use of ICT (19, 21, 36–38). Poor overall mental health and higher intensity of symptoms of psychosis were described as significant barriers to ICT use within eight studies (19, 31–33, 38–41). Authors from a study from Taiwan exploring the relationship between sleep quality, social functioning, self-stigma, and problematic smartphone use suggested that problematic use of ICT was a barrier to further ICT use by participants with psychotic disorders (42). These authors defined problematic use of ICT as excessive use of smartphones generating negative consequences for individuals’ health.

Secondly, the level of ICT knowledge and interest in using ICT were positioned as moderators of ICT use and access. The results of four studies highlight that individuals who lack interest in ICT or who prefer face-to-face interaction are less likely to use ICT (19, 21, 31, 33). Increased digital literacy was associated with increased ICT use in three studies (33, 34, 43).

Age was suggested to be a significant moderating factor of ICT use. Specifically, in 8 studies, the results suggest that increased age is directly associated with reduced ICT use (31–34, 39, 44–46). Other social determinants of health such as relationship status, race, gender, socioeconomic status, education level, rural versus urban environments, and living with HIV, were also positioned as significant moderators of ICT use and access in these studies. Specifically, being married or partnered (46), Caucasian (44), female (44), higher income (19, 40, 46), having higher education (34, 39, 40, 45), living in urban environments (34, 46), and living with HIV (46) were highlighted as being associated with increased ICT use and access. The authors of a study conducted in the United States explored social “connectedness” among patients with schizophrenia, if ICT interferes with mental illness, and if patients envision ICT as being involved in their treatment. The authors suggest that individuals identifying as women were more likely than individuals identifying as men to express that ICT has the potential to exacerbate paranoia, while individuals identifying as men were more likely than individuals identifying as women to endorse that ICT worsened auditory hallucinations (44). Lastly, authors from one study conducted in the United States described the development of a smartphone illness self-management system for people with schizophrenia and reported that a the majority of participants with psychotic disorders used affordable phone plans due to low income (37).

### Potential benefits of ICT

Potential benefits of ICT included self-management of mental illness, impacts to mental health, empowerment, equity, and hope for the future, influence on social interaction and social support, accommodations and accessibility considerations, and potential growth of ICT use with support. Fourteen papers were coded in this category.

Persons living with psychotic disorders who were using or intending to use ICT for self-management supports was reported in seven studies (19, 37, 44–48). Participants with psychotic disorders were noted to use ICT broadly for this purpose, from seeking information related to self-management online (19, 45–47), to sharing recovery stories (47, 48) and communicating with their healthcare team (44). Participants with psychotic disorders in five of the included studies were described as wanting further digital self-management supports to enhance their recovery (19, 37, 44–46). In one study conducted in the United States, the authors describing the development of a smartphone illness self-management system for people with schizophrenia, clinicians reported they believed clients living with psychotic disorders could learn to use a mobile device for self-management (37).

The results of four studies suggested significant positive impacts to mental health, empowerment, equity, and hope for the future through using and accessing ICT (19, 36, 46, 47). For example, Schrank and colleagues conducted 26 semi-structured interviews to investigate the nature and subjective consequences of health-related Internet use among participants with schizophrenia (19) and reported the Internet is an influential source of illness-related information for people with psychotic disorders. Naslund and colleagues conducted qualitative analysis of 19 YouTube videos and their associated 3044 comments to explore how individuals with SMI use YouTube to interact with each other (47). Increases to self-esteem and affect (36), positive behavior changes (19), reductions in negative affect and paranoia (36), and more hope for the future (47) were many of the identified benefits to mental health. One survey of 165 participants in South Africa that focused on access to, use and perception of ICT reported that 71.5% of participants described Internet use as beneficial for their mental health (46). Further, Schrank and colleagues reported multiple benefits including the opportunity to anonymously telling one’s recovery story, positive changes to relationships with participants’ doctors (19), and perceived online equality (19).

Two studies suggested potential for growth of ICT use with support (21, 37). Ben-Zeev and colleagues (38) surveyed 904 participants across the United States with schizophrenia or schizoaffective disorder regarding their use of mobile devices and interest in mHealth services, as well as 8 practitioners about their attitudes and expectations from an mHealth intervention. These researchers incorporated consumer and practitioner input to produce an mHealth intervention, and had 12 consumers participate in laboratory usability sessions (37). The participants living with psychotic disorders learned how to use a mobile device and self-management system after a brief tutorial; However, no information was provided about who provided the training, for how long, and whether a pedagogical approach was used. A pilot study by Beebe and colleagues (37) examined the feasibility and acceptability of cell phone use among 10 individuals with schizophrenia spectrum disorders in the United States; participants were provided with an activated cell phone for 5 months and trained nurses contacted the participants weekly (21). The authors suggested participants with psychotic disorders could retain cell phones for extended periods of time and engage with this technology.

Accommodations and accessibility considerations with ICT uptake were reported in two studies (37, 49). Peer-to-peer facilitators supported the uptake of digital interventions among participants living with psychotic disorders (49). Participants with psychotic disorders in a second study also reported seeing value in using images and visual aids in app use (37). Service users being involved in digital intervention design also showed higher rates of acceptability (49).

Positively influencing social interaction and social support was another suggested benefit of using and accessing ICT in eight studies (19, 31, 32, 37, 40, 44, 47, 49). In four studies, it was suggested that participants living with psychotic disorders used or wanted to use ICT with the specific goal of engaging in social support (31, 32, 40, 44). Other studies suggested that participants with SMI were less socially isolated (37, 40, 44, 47), had more community involvement (32), and engaged in formal peer support (19, 49) because they used ICT.

### Potential risks of ICT

Potential risks reported in the studies included: challenges with keeping devices; concerns with anonymity, security, and privacy; and direct negative impacts to mental health. There are differing viewpoints about the extent to which patterns of ICT use and access are problematic and whether harm is possible or inevitable; potential problematic ICT use, and lack of evidence that ICT leads to worsening mental health were also reported in the research. Nine papers were coded in this category.

Two studies described challenges related to safety and security of the technology due to the frequent financial precarity of people with psychotic disorders (21, 37). Authors of one study that provided 10 participants with activated cell phones suggested participants had challenges keeping devices due to having their cell phone stolen or participants selling their cell phone to obtain illegal substances (21). One mixed methods study that interviewed clinicians regarding their beliefs about participants with psychotic disorders and ICT use reported that over half of the clinicians in the study believed participants/clients would sell their ICT device, with some thinking the participants might break it for unspecified reasons (37).

Anonymity, security, and privacy were other challenges identified in three studies (32, 33, 47). Authors of a systematic review of 13 studies in the United Kingdom reported that one of their included studies specifically noted that ICT can lack anonymity, which could be potentially overlooked by participants with SMI, leading to associated risks of unintended disclosure (32, 47). Concerns about privacy, personal security, and the inability to verify the identity of digital contacts were other risks highlighted by the authors of this same systematic review in 3 of the 13 included articles (32), as well as the authors of a study surveying 367 adults with SMI about ICT use, access, and changes in mental and physical health since the beginning of pandemic restrictions (32, 33).

Direct negative impacts to mental health were reported in five studies and included: worsening psychotic symptoms and adverse emotional responses (19, 32), paranoia and negative affect (36), and general decline in mental health (33). For example, in a study using an experience sampling method to evaluate surveys completed by 44 participants with and without psychotic disorders over a six-day period, social media use significantly predicted low mood and posting about feelings, while venting online significantly predicted low mood, self-esteem and high paranoia (36). Authors conducting a survey with 367 adults with SMI suggested that using the Internet “a lot” during the pandemic was associated with a self-reported decline in mental health (33). Although not an intervention study, authors of one study reported that 8 clinicians recruited to represent a range of specializations and service models expressed general beliefs and concerns that mobile devices would increase psychotic symptoms in participants living with psychotic disorders (37).

Concerns regarding potential problematic ICT use were identified in four studies (19, 32, 42, 47). Problematic smartphone use, or excessive use of ICT leading to potentially negative health consequences, was suggested to be related to poor sleep quality, self-stigma, and poorer social functioning by authors of a study surveying participants with schizophrenia at 5 different timepoints with three-month intervals between each timepoint (42). Potentially harmful online interactions were found to be another concern related to ICT overuse in 3 articles in a systematic review of 13 articles and qualitative analysis of 19 YouTube videos with 3044 associated comments (32, 47). Critical attitudes towards psychiatric medication were highlighted as a concern when participants engaged in ICT use (19). In this same study, participants with schizophrenia cited fear of Internet addiction as a potential risk of using ICT (19).

Two studies suggested a lack of evidence that ICT leads to worsening mental health (32, 44). Jakubowska et al. noted that six of the studies included in their systematic review reported that there is a lack of evidence that ICT leads to worsening mental health (32). Another quantitative non-experimental study surveying inpatients and outpatients with schizophrenia on ICT use and associated attitudes reported that more than half of its 80 participants using ICT did not feel that technology worsened their mental health (44).

## Discussion

In this scoping review, we explored that literature to ascertain what has been written about how people living with psychotic disorders access or use ICT for social connection and community participation, as well as the barriers and facilitators and the risks and benefits to using ICT for people living with psychotic disorders. We identified 19 articles that met the inclusion criteria. Through our analysis, we identified trends in ICT use among participants with psychotic disorders, moderators of ICT use, potential benefits of ICT, and potential risks of harm related to ICT use. The included articles were primarily from high income countries and most studies provided low level evidence. However, the results of this review suggest that ICT use and access is complex, multifaceted, and can be an important and influential tool for participants living with psychotic disorders with potential for recovery-based mental health services.

There are similarities in ICT use between participants with psychotic disorders and other populations reported in the literature (50–52), with the reasons and goals of ICT use and access being similar (21, 31–34, 36). Though the general use of the Internet and other forms of ICT are growing (53), social determinants of health as well as social connection factors impact their use in the general population (54). For example, in da Costa and colleagues’ quantitative non-experimental study exploring the views and interests of participants with psychotic disorders on different formats of volunteering (31), there was a significant association between interest in getting face-to-face volunteering with loneliness and quality of life as significant predictors of this. In addition, digital volunteering interest was predicted by age and years since diagnosis (31). This is similar to other populations and how they use ICT, such as (50, 51). In Spanakis and colleagues’ quantitative non-experimental study on identifying the extent to which people with SMI have been using the Internet, it was identified that most participants owned a digital device and had access to the Internet from home (33). The majority of participants with schizophrenia in Fernandez-Sotos’ and colleagues’ quantitative non-experimental study reported they used a smartphone daily (34).

Patterns of ICT use are impacted by cognitive impairments, psychiatric symptoms, as well as financial barriers to accessing technology. Improvements to digital literacy may impact comfort level with technology and its learning curve, particularly for older adults. Several benefits to the use of ICT were reported in the reviewed literature; these were related to self-management of mental illness, and evidence for positive impact on mental health, empowerment, equality, and hope for the future, influence on social interaction and social support, improvements in accommodations and accessibility considerations, and potential growth of ICT use with support. Finally, potential risks of harm related to ICT use and access reported in the included studies involved challenges with keeping devices, concerns with anonymity, security, and privacy, direct negative impacts to mental health, and potential problematic ICT use. There were also studies that reported a lack of evidence that ICT leads to worsening mental health. The identified risks are also somewhat similar to the general population; though ICT offers access to information and facilitates communication, there are several risks related to security, scams, Internet addiction, and possible direct impacts to mental health (55). This is in direct contrast with a systematic review by Ahmed and colleagues, which suggested that there are small but significant positive associations between social media use, depression, and anxiety (56). The authors of this systematic review also found that problematic social media use was positively associated with depression, anxiety, and sleep problems, with a negative association with wellbeing (56). Therefore, it is imperative that the benefits and risks to ICT use and access be carefully balanced when applied to populations with greater vulnerabilities.

The results of this scoping review lay the foundation not only for further research related to how people with psychotic disorders access or use ICT for social connection and community participation as well as identifying the risks, benefits, barriers, and facilitators to ICT use; the results also provide direction for clinical assessment and intervention. What remains entirely absent from the existing literature is the development and evaluation of any clinical intervention to directly boost ICT use and access. For example, if it is true that there are moderators to ICT use and access, then there should be work done to address these to optimize these factors. Such an intervention should be co-designed in collaboration with people who might benefit from it, including people living with psychotic disorders. We echo the inherent definition of occupational justice and its application to this topic; all individuals, including people with psychotic disorders, have the right to engage in meaningful and diverse occupations, and to have equal opportunities to reach their full potential (57). Failure to adequately address barriers to ICT use and access presents an issue of equity and justice and could have a negative impact on overall community participation and access to social connections and other meaningful activities. There is a need to address the barriers faced by people with psychotic disorders, while simultaneously addressing the documented risks and potential negative health effects of problematic ICT use among populations such as youth (56).

### Implications and recommendations

This scoping review is the first known attempt to synthesize data regarding what has been written about people living with psychotic disorders and how this population uses ICT for social connection and community participation. It is clear that in recent years, there has been a surge of ICT with a number of potential uses. What is perhaps less clear is that people with psychotic disorders are sometimes being left behind the general population as a result of the moderating factors discussed in this paper; this is an issue of equity and justice. Therefore, it is essential that future research not simply investigate differences in ICT use and access, moderating factors of use and access, benefits, and risks, but focus upon how to include this population equitably in this critical occupation through direct intervention. There was only one study included in this scoping review involving components of an intervention related to increasing cell phone use (21); it had a small sample size of 10 participants and unfortunately also reported that some data were missing.

### Strengths and limitations

Strengths of this scoping review included the systematic approach to searching the literature (multiple databases, comprehensive search terms, no restrictions on language), and by including articles published since 1992. The comprehensiveness of the search was limited by including only peer reviewed academic journal articles. Although the first author coded, categorized all of the codes, and met with the last author to finalize the results, the data could have been interpreted differently by others. It is also important to note that the first author’s positionality as an occupational therapist and case manager with individuals living with psychotic disorders may have impacted the interpretation of the findings.

All studies noted potential benefits of ICT, even though some risks were also noted. This may be because there is an benefit to this population or because of a publication bias, where studies with no significant impact are often not published (58). Another potential limitation is that only one study included the perspective of healthcare providers; further research on the perspective of clinicians may be useful.

## Conclusion

The results of this scoping review underscore differences and similarities between people with psychotic disorders and other populations, moderators of ICT use and access, potential benefits of ICT, and potential risks of harm of ICT. Many of the moderating factors identified within this review could plausibly be modified through direct intervention, such as opportunities to gain ICT knowledge and interest, and education. Thus, the use of and access to ICT by this population should be comprehensively evaluated to optimize the potential benefits and minimize the potential risks. ICT can be empowering and enabling, and has the potential to shape the nature of service, support, and care people with psychotic disorders receive in line with principles of recovery.

## Data Availability

The original contributions presented in the study are included in the article/**Supplementary Material**. Further inquiries can be directed to the corresponding author.
