# Correction: How do people living with psychotic disorders access and use information and communication technology: a scoping review

**DOI:** 10.3389/fpsyt.2026.1982653

**Published:** 2026-09-09

**Authors:** Jaclin Vozza, Rebecca Ripco, Sandra Moll, Evelyne Durocher, Rebecca Gewurtz

**Affiliations:** 1 School of Rehabilitation Science, McMaster University, Hamilton, ON, Canada; 2 Schizophrenia Outpatient Clinic, St. Joseph’s Healthcare Hamilton, Hamilton, ON, Canada

**Keywords:** serious mental illness, technology, social connection, community participation, psychotic disorders

An incorrect **Funding** statement was published. The name of the funder, “the Inclusive Design for Employment Access Social Innovation Laboratory, and its funding source, the Government of Canada’s New Frontiers in Research Fund (NFRF)”, were omitted. The correct **Funding** statement reads:

“The author(s) declare that financial support was received for the research and/or publication of this article. The first author received financial support to carry out this research from the Inclusive Design for Employment Access Social Innovation Laboratory, with funding from the Government of Canada’s New Frontiers in Research Fund (NFRF) [NFRFT-2020-00339].”

The original version of this article has been updated.

