# Supplementary material for: How do people living with psychotic disorders access and use information and communication technology: a scoping review
Source: Front Psychiatry. 2025 Aug 7;16:1639348. doi: 10.3389/fpsyt.2025.1639348 (PMC12367688; doi:10.3389/fpsyt.2025.1639348)
Supplement: Supplementary file 1 [file Table1.docx]

**Appendix A**

**Search Strategy Example: MEDLINE Search Terms**

(exp "Schizophrenia Spectrum and Other Psychotic Disorders"/ OR SMI.tw. OR Serious-mental-illness*.tw. OR serious-mental-disease*.tw. OR Severe-mental-illness*.tw. OR severely-mentally-ill*.tw. OR persistent-mental-illness*.tw. OR chronic-mental-illness*.tw. OR chronically-mentally-ill*.tw. OR schizophren*.tw. OR schizoaffective*.tw. OR psychos*.tw. OR psychot*.tw.) AND (Youtube.tw. OR facebook*.tw. OR smartphone*.tw. OR mHealth.tw. OR eHealth.tw. OR website*.tw. OR GPS.tw. OR social media*.tw.OR telemet*.tw. OR telemed*.tw. OR telepsychiat*.tw. OR telehealth.tw. OR telecare*.tw. OR telemental-health*.tw. OR ehealth.tw. OR mhealth.tw. OR mobile*.tw. OR mobile-health*.tw. OR mobile-technolog*.tw. OR mobile-phone*.tw. OR cellular-phone*.tw. OR cellphon*.tw. OR textmessag*.tw. OR text-messag*.tw. OR short-message-service*.tw. OR SMS.tw. OR internet-health*.tw. OR internet*.tw. OR online*.tw. OR social-media*.tw. OR tablet*.tw. OR bluetooth*.tw. OR avatar*.tw. OR GPS.tw. OR global-positioning-devic*.tw. OR global-positioning-system*.tw. OR exp "Geographic Information Systems"/ OR geographical-information-system*.tw.OR exp Telecommunications/ OR telecommunic*.tw. OR virtual-real*.tw. OR VR.tw. OR exp "Virtual Reality"/ OR website*.tw. OR web-base*.tw. OR web-brows*.tw. OR remote-consult*.tw. OR remote-sens*.tw. OR artificial-intelligenc*.tw. OR AI.tw. OR user-computer-interfac*.tw. OR computer-simulat*.tw. OR speech-recognition-softwar*.tw. OR computer-assisted-therap*.tw. OR computer-base*.tw. OR wireless-technolog*.tw. OR remote-sensing-technolog*.tw. OR exp "Medical Informatics Applications"/ OR informatics-applic*.tw. OR exp Telemedicine/ OR exp "Text Messaging"/ OR exp "Social Media"/ OR exp "User-Computer Interface"/ OR exp "Medical Informatics Applications"/ OR exp "Computer Simulation"/ OR exp "Web Browser"/ OR exp Telemetry/ OR exp "Game Theory"/ OR exp Smartphone/ OR exp "Computers, Handheld"/ OR handheld-computer*.tw. OR (computer*.tw. AND palm-top*.tw.)OR PDA.tw. OR (personal*.tw. AND digital-assist*.tw.) OR (tablet*.tw. AND computer*.tw.) OR exp Microcomputers/ OR personal-comput*.tw. OR PC.tw. OR exp Internet/ OR exp "Artificial Intelligence"/ OR exp "Mobile Applications"/ OR exp "Online Systems"/ OR exp "Cell Phone"/ OR exp "Therapy, Computer-Assisted"/ OR exp Software/ OR exp Crowdsourcing/ OR exp "Social Networking"/ OR exp Blogging/ OR exp Robotics/) AND (exp "social connection"/ OR "social connect*".tw. OR exp "community participation"/ OR relation*.tw. OR "social support*".tw.) OR exp "social networking"/ OR exp "social participation"/ OR exp "community support"/

**Appendix B**

**Data Extraction Template**

| **Author(s), (year), country** | **Objective/Aim** | **Study**  **Design** | **Participants/Sample population** | **Information about Accessing and Using ICT** | **Additional Considerations** |
| --- | --- | --- | --- | --- | --- |
|  |  |  |  |  |  |
|  |  |  |  |  |  |

**Appendix C**

**Characteristics of Included Studies Table**

| **Citation** | **Country** | **Aim of Study** | **Study Design** | **Participants/ Sample Population** | **Sample Size** | **Information about Using and Accessing ICT** |
| --- | --- | --- | --- | --- | --- | --- |
| Haker H, Lauber C, Rössler W. Internet forums:: a self-help approach for individuals with schizophrenia? ACTA PSYCHIATRICA SCANDINAVICA. 2005;112(6):474-7. | Switzerland | To study if and how online self-help forums for individuals with schizophrenia are used | Qualitative research | - 12 international schizophrenia forums; 1200 postings; 100 most recent postings in each forum - 58.7% over all forums self-reported mental illness; 81% schizophrenia/psychosis, 19% anxiety or affective disorders - Proportion of relatives was 28.4%; 12.9% non-related individuals and friends | 576 forum posts | - Self-help mechanisms: disclosure of personal experiences (47.7%), providing information (42%), request for information (27.8%), expression of empathy (20.8%), gratitude (9.3%), friendship (0.7%) - Fields of interest: discussions about daily problems of the illness like symptoms (33%), medication (33%) and emotional involvement with the illness (33%). Non-pharmacological therapies and information about resources were other topics of interest (25% of postings) - Disclosure of illness: 57.9% vs. 34.7% written by affected individuals; relatives contributed more to provide information on questions asked by others (50.6% vs 35.2%) - Affected users were significantly more interested in describing symptoms 41.3% vs. 27.9%), discussing medication (37.3% vs. 21.7%) and vocational issues (11.2% vs. 5.5%) - Friends and relatives were significantly more often exchanging information and resources (27.7% vs. 17.6%), talking about social network (31.1% vs. 15.4%), substance use (7.9% vs. 3.7%) and legal issues (7.5% vs. 2.8%) |
| Beebe LH, Smith K, Bennett C, Bentley K, Walters AB, Hancock B, et al. Keeping in touch Cell Phone Use in People with Schizophrenia Spectrum Disorders. JOURNAL OF PSYCHOSOCIAL NURSING AND MENTAL HEALTH SERVICES. 2010;48(4):32-7. | USA | To examine the feasibility and acceptability of cell phone use in individuals with schizophrenia-spectrum disorders | Non-randomized experimental study | - 8 participants with schizoaffective disorder - 2 clients with schizophrenia - 50% men, 50% women - 80% Caucasian, 20% African American - 50% living alone, 40% with family, 10% with caregiver - 10% grade school education, 40% some high school, 30% high school graduate - *Some data are missing | 10 | - Average of four attempts were made for each completed telephone contact - Average length of call was 5.8 minutes (range of 2-14 minutes) - Number of missed calls increased with time  - One participant lost interest in the phone and returned it to investigators - Participants reported problems related to operating the phone and forgetfulness - The study showed that many individuals with SMI are able to retain cell phones for extended periods of time and are willing to answer calls from caregivers  - One participant had phone stolen from their group home after the third week of use  - One participant sold their phone within four days of receiving it and used the money to but illegal substances |
| Spinzy Y, Nitzan U, Becker G, Bloch Y, Fennig S. Does the Internet offer social opportunities for individuals with schizophrenia? A cross-sectional pilot study. PSYCHIATRY RESEARCH. 2012;198(2):319-20. | Israel | To compare Internet usage as a social outlet between psychotic and non-psychotic and control groups | Quantitative non-experimental | - 143 participants with psychotic disorders: average age 43.83 - 118 with affective or anxiety disorders: average age 47.34 - 100 healthy volunteers: average age 36.52 | 361 | - Psychotic group had significantly fewer friends than other groups - A higher percentage of psychotic group wished to establish social connections - Psychotic group had a lower income and education level than other groups - Severity of illness was significantly correlated with fewer social connections and lower Internet accessibility and usage - Psychotic group had significantly lower Internet accessibility and reported less web-browsing time compared to the non-psychotic group - Psychotic group had a higher correlation of creating virtual relationships and real-life relationships through the Internet- |
| Grossman MJ, Woolridge S, Lichtenstein S, McLaughlin D, Auther AM, Carrion RE, et al. Patterns and perceptions of face-to-face and digital communication in the clinical high risk and early stages of psychosis. PSYCHIATRY RESEARCH. 2020;284. | Canada & USA | To examine characteristics of face-to-face and digital communication in youth at clinical risk for psychosis or in the first episode of psychosis, as well as age-matched community comparisons | Quantitative non-experimental | - 53% of Clinical High Risk (CHR) group and 53% of community controls were male; 71% male in First Episode Psychosis (FEP) group - FEP group average age 23.64; CHR average age 17.26; community controls average age 21.09 | 127 | - Community controls engaged in significantly more frequent communication than CHR and FEP participants - Community controls were significantly more satisfied with communication than CHR and FEP participants - Community controls had higher satisfaction with face-to-face than digital communication - Community controls had significantly greater challenges with digital than face-to-face communication - Significantly more challenges with communication in FEP and CHR groups than community controls - Satisfaction was positively associated with frequency of contact in both communication formats in clinical sample - Number of challenges reported were unrelated to frequency of contact for clinical sample and were negatively associated with level of satisfaction in both formats |
| Naslund JA, Grande SW, Aschbrenner KA, Elwyn G. Naturally Occurring Peer Support through Social Media: The Experiences of Individuals with Severe Mental Illness Using YouTube. PLOS ONE. 2014;9(10). | USA | 1) To explore the phenomenon of individuals with serious mental illness uploading videos to YouTube and posting and responding to comments as a form of naturally occurring peer support 2) To consider the potential risks and benefits of self-disclosure and interacting with others on YouTube | Qualitative research | - Publicly available videos that had been extensively viewed with the following search terms: "mental illness", "schizophrenia", "schizoaffective disorder", or "bipolar disorder"; also reviewed recommended video lists to identify additional videos - 8 self-identified as being diagnosed with bipolar disorder, 7 with schizoaffective disorder, 4 with schizophrenia - Common topics included managing illness symptoms, challenges and benefits of seeking treatment, coping strategies for day-to-day concerns, efforts to reach out and help others with similar conditions, and personal stories about life goals, interests, and future ambitions - Average 19786 views (range of 5433 to 88654) - Videos had been on YouTube between 87 and 1798 days - 15 videos posted by young adults (18-35) and 4 by middle-aged adults (36-55) - 3044 comments posted; 5% were flagged as inappropriate | 19 videos uploaded by 19 different individuals | - Lack of anonymity and associated risks of being identified as an individual with SMI seemed to be overlooked by commenters and video authors - 5% of YouTube comments viewed were flagged as inappropriate because they were either spam, advertisements, derogatory, or discriminatory - Major theme of minimizing a sense of isolation and providing hope - Major theme of finding support through peer exchange and reciprocity - Major theme of coping with the day-to-day challenges of SMI - Major theme of learning from shared experiences of medication use and seeking mental health care |
| Biagianti B, Quraishi SH, Schlosser DA. Potential Benefits of Incorporating Peer-to-Peer Interactions Into Digital Interventions for Psychotic Disorders: A Systematic Review. PSYCHIATRIC SERVICES. 2018;69(4):377-88. | USA | To examine the feasibility, acceptability, and preliminary efficacy of recent digital interventions in order to identify strategies to maximize benefits of online peer-to-peer communications for persons with psychotic disorders. | Systematic review | 8 studies included; 3 RCTs, 1 usability testing comparison, 4 uncontrolled pilot studies | Not explicitly reported | - Technology supporting peer-to-peer communication varied greatly across studies - When peer-to-peer interactions were moderated by facilitators, retention, engagement, acceptability, and efficacy were higher than for interventions without facilitators - Individuals with psychotic disorders were actively engaged with moderated peer-to-peer communications - Studies involving service users in intervention design showed higher rates of acceptability - Individuals with psychotic disorders showed improvements in perceived social support with use of peer-to-peer interaction technology |
| Ben-Zeev D, Kaiser SM, Brenner CJ, Begale M, Duffecy J, Mohr DC. Development and usability testing of FOCUS: a smartphone system for self-management of schizophrenia. Psychiatric rehabilitation journal. 2013;36(4):289-96. | USA | To describe the staged approach that led to the development of FOCUS, a smartphone system specifically designed to support self-management of illness among people with schizophrenia. | Quantitative non-experimental & qualitative | - Individuals receiving care and practitioners at a large psychiatric rehabilitation agency in Chicago - 904 clients with schizophrenia or schizoaffective disorder with a mean age of 47; 68% men, 61% African American, 37% Caucasian, 5% Hispanic; 34% less than high school education, 38% high school diploma, 28% some post-high school education; 74% had an income of $10 000 or less annually - 8 practitioners working in mental health - 12 individuals diagnosed with schizophrenia or schizoaffective disorder; mean age of 45, 67% men, 75% African American, 8% Caucasian, 17% Hispanic; 50% less than high school diploma, 17% high school diploma, 33% some post-high school education | 904 clients for survey; 8 practitioners; 12 clients for usability testing | - 63% the surveyed clients of client participants owned a mobile device; 91% used it for talking, followed by texting (31%) and Internet access (13%); daily use reported by 58% - 75% of the usability testing clients currently owned and used a mobile device, with only one owning and using a smartphone - Finances: 22% reported using a month-to-month plan, 25% reported using "government minutes" (federal "Lifeline" program that helps low-income individuals pay for mobile phone service in the US - Difficulty operating a mobile device: practitioners believed that clients would have difficulty with this - All usability testing participants were able to learn to use the mobile device and illness self-management system following a brief tutorial - All participants saw value in images and visual aids - Many respondents indicated they would be interested in mHealth services to their mobile device - Over half the practitioners believed the clients would sell the device, with some thinking they might break it - One practitioner believed clients might experience an increase in psychosis because of the device - Practitioners believed mobile devices could be helpful for clients who are "hard to reach" - Practitioners believed clients could use a mobile device for self-management of their illness - Many usability testing clients had difficulty understanding abbreviations and longer words used in training |
| Schrank B, Sibitz I, Unger A, Amering M. How patients with schizophrenia use the Internet: qualitative study. J Med Internet Res. 2010;12:e70. | Austria | To investigate the nature and subjective consequences of health-related Internet use among patients with schizophrenia | Qualitative research | - 54% male; 46% female - Age range of 18-52 - 77% reported their main diagnosis to be schizophrenia; 23% schizoaffective disorder - Age of first onset of illness was between 11-44 - All but 2 participants hospitalized at least once - 62% single, 23% partnered, 15% separated/divorced - 15% unemployment benefit, 4% social welfare benefit, 12% student, 42% disability pension, 19% employed, 8% other - 35% living in own household with partner/family, 35% living alone, 8% living in flat share, 12% parents' household, 12% supported housing - 4% no formal education, 19% compulsory schooling, 27% primary education, 50% secondary education (including post-secondary) - General Internet use ranged from sporadic to several hours a day - Majority reported having searched for illness-related information on Internet | 26 | - Information from the Internet had the potential to significantly change the relationship with attending doctors - Participants wanted reliable information and recommendations from their doctors about what sites to access - Patients wanted to be able to access their doctors online  - Interviewees often had problems defining the issues they had been interested in - Lack of access to a computer, financial issues, fear of Internet addiction, preference for other sources of information, fear of viruses, and expectation of low quality of Internet information were cited as reasons against using the Internet  - Illness related barriers: stimulus overflow and inability to deal with abundance of information, concentration issues, lack of energy and depressive symptoms, paranoid ideas and fear of symptom provocation, and wish to distance oneself from illness-related topics as part of the recovery process - Skepticism about the quality and credibility of Internet information  - Interviewees reported looking for information about their illness - Internet has anonymity and egalitarianism - Positive effects: empowerment, anonymously telling one's own story, peer support, information, behaviour changes - Negative effects: worsening symptoms and aversive emotional responses, critical attitude towards medication |
| Sharma-Misra S, Maru M, Tomita A, Paruk S. Access and use of digital technology by patients with psychosis at a hospital in South Africa. South Africa J Psychiat. 2023;29(1):a2151. | South Africa | To describe the access to, use and perception of ICT in people with schizophrenia and other psychotic disorders | Quantitative non-experimental | - Mean age of 41 - 45.5% female, 54.5% male - 54.5% single, 32.1% married, 13.3% divorced, widowed or separated - 77% no tertiary education, 23% had tertiary education - 31.5% R0-R4999 income per month, 33.3% R5000-R9999 per month, 35.2% R10 000 or more - 37.6% employed, 29.1% unemployed, 33.3% on disability grant - 93.3% urban, 6.7% rural - 12.1% living with HIV - 52.1% alcohol use in the past 3 months - 38.2% cannabis use in the past 3 months | 165 | - 83% of participants had used Internet in the last 3 months - 93.9% owned an electronic device to access the Internet - Those who were married were 21x more likely to use the Internet in the last 3 months when compared to those who were divorced, separated, or widowed - Higher income group had higher odds of using the Internet - Those living in rural settings had 93% lower odds of using the Internet - Living with HIV was associated with 5x the odds of more frequent Internet use - 1 year age increase was associated with 5% lower odds of more frequent Internet use and 7% lower odds of using an app - Higher income group had higher odds of seeking mental health information on the Internet - 64.8% used the Internet to seek information about mental health - 71.5% agreed that Internet use was beneficial to mental health and 81.4% wanted an app to help cope with SMI |
| Tanriverdi Olug S, Balaban OD, Gul O, Altin MO. The Relationship of Internet, Social Media, and Related Technology Use with Disease Severity and Functionality in Individuals with Serious Mental Disorders. Noropsikiyatri Arsivi. 2024;61(1):3-10. | Turkey | 1) To assess the utilization of social media and related technologies 2) To examine the relationship between social media use and the severity of mental health disease and functionality 3) To compare social media use between two patient groups 4) To gain insight into the thoughts of mental health patients regarding the benefits and risks of social media use by investigating the social media use of a sample of individuals diagnosed with bipolar disorder or schizophrenia spectrum disorders | Quantitative non-experimental | Psychotic group:  - 54.7% with schizophrenia, 37.3% with psychosis NOS, 8% schizoaffective disorder - 33.3% female, 66.7% male  - 22% working, 72% unemployed, 6% irregular employment - 22.7% married, 63.3% single, 14% divorced/widowed - Average years of education 7.44 - Average age 41.45  Bipolar group: - 50.7% female, 49.3% male - 30% working, 64.7% unemployed, 5.3% irregular employment - 48.7% married, 37.3% single, 14% divorced/widowed - Average years of education 8.24 - Average age 40.96 | 300 | - Being married and female were higher in bipolar patients compared to psychosis group - 65.3% use the Internet, 59.7% use social media, 50.7% use a computer, 80.7% use mobile phones, 36.3% use email, 53.7% text - Use of mobile phones, social media, smartphones, texting, and email was significantly higher in bipolar group than psychosis group - Patients who used social media were significantly younger and had a significantly higher education level than those who did not use social media - Significantly higher scores on global impression scale for those who did not use social media - FAST scales were significantly higher in psychosis group for those that did not use social media compared with those that did |
| Miller B, Stewart A, Schrimsher J, Peeples D, Buckley P. How connected are people with schizophrenia? Cell phone, computer, email, and social media use. Psychiatry Research. 2015;225:458-63. | USA | 1) To determine how connected patients are with schizophrenia 2) To determine if technology interferes with patients' illness 3) To determine if patients with schizophrenia envision technology as being part of their treatment | Quantitative non-experimental | - Average age was 41 - 51% male - Average age of first psychiatric hospitalization was 23 - 56% African descent, 31% Caucasian, 11% other - 63% single, 14% married, 23% widowed/divorced/separated | 80 | - 73% of participants own or have access to a cell phone; 56% use cell phone to text; 5% have sent/received a text to/from a doctor; 54% have access to a computer; 48% have access to the Internet; 48% have an email account; 11% have sent/received email message to/from a doctor - 43% use texting at least daily, 20% use a computer daily, 17% check email at least daily, 27% use social networking sites at least daily - 53% denied using social media sites; most popular were Facebook (39%), Google Plus (16%), MySpace (10%), Twitter (6%), and Instagram (1%) - Age: participants using a computer, email, and social networking were significantly younger than non-users; age was significantly negatively correlated with endorsing that computer or email helps them interact with others - Sex: significantly fewer males than females are current text message users; females significantly more likely to say computer or email make them feel paranoid or suspicious while males said that voices would be worse - Race: Caucasians were significantly more likely than those of African descent to endorse that social networking helps interactions with others - More than half of participants that used computer, email, and social networking disagreed that technology worsened their mental health - More than half of participants that used computer, email, and social networking agreed that technology helps them interact and socialize more - 37% expressed interest in an online chat with a peer group of persons with mental illness - 58% of participants were interested in text reminders of appointments, and at least 40% were interested in text reminders to take medications or to inquire about symptoms, medication side effects, or other problems - 43% interested in email reminders about appointments and 50% were not interested in email reminders to take medications |
| Thomas N, Alfred M, Foley F, Lindblom K, Lee S. Are people with severe mental illness ready for online interventions? Access and use of the Internet in Australian mental health service users. Australasian Psychiatry. 2017;25(3):257-61. | Australia | 1) To understand those with SMIs' access to, and confidence with using, the Internet 2) To understand current views on using online resources as part of mental healthcare | Quantitative non-experimental | - Majority of sample had schizophrenia-related disorder - Age: 1% 18-24, 27% 25-34, 33.3% 35-44, 22.2% 45-54, 16.2% 55-64 - 57% male - Education: less than high school 44.3%, high school 13.4%, certificate/diploma 13.4%, degree 28.9% - Employment: unemployed 70.4%, volunteering 10.2%, part-time 5.1%, full-time 5.1%, student 9.2% | 100 | - 86.7% had a mobile phone - 25.5% no access to a computer - 83.7% no access to tablet - 33.7% no access to Internet excluding mobile phone - 42.9% no access to Internet on mobile phone - 28.9% no email address - 48% used social media - 33% members of forums - 74% confident using Internet, and 70% confident using email independently or with occasional assistance - Age: Internet access significantly decreased with age and increased with level of education - 73% willing to receive email from healthcare team, 82% willing to receive texts from healthcare team - 65% willing for a mental health care worker to use a tablet computer with them during appointments to look at materials on mental health |
| Berry N, Emsley R, Lobban F, Bucci S. Social media and its relationship with mood, self-esteem and paranoia in psychosis. Acta Psychiatrica Scandinavica. 2018;138(6):558-70. | UK | To explore in real time the impact of social media use on mood, self-esteem, and paranoia in people who experience psychosis | Quantitative non-experimental | - Mean age of 33.7 for participants with psychosis; 37% male, 63% female, 11% working part-time, 11% volunteering, 11% student, 68% unemployed; 4% Asian, 95% White British; 26% high school graduate, 47% college, 16% some university, 11% undergraduate degree - Mean age of 35.4 for non-clinical sample; 44% male, 56% female, 52% working full-time, 12% working part-time, 28% student, 8% unemployed; 4% Asian, 84% White British, 8% White other, 4% mixed race; 16% high school graduate, 16% college, 16% some university, 40% undergraduate degree, 12% post-graduate degree | 44; 25 non-clinical and 19 with schizophrenia-spectrum | - Three participants in the clinical group and one participant in the non-clinical group borrowed a smartphone for the duration of the study - Participants in the clinical group reported lower use of social media use compared with the non-clinical group - The impact of social media use did not differ between participants with and without psychosis - For some reason, social media use frequency was lower in people with psychosis - Higher self-esteem and higher positive affect in non-clinical group compared with clinical group when using social media - Clinical group had higher level of negative affect and paranoia compared with non-clinical group - Content posting predicted negative affect and paranoia - Social media content consumption predicted negative affect - Posting about daily activities led to increase in positive affect and self-esteem - Posting about feelings led to increases in negative affect, paranoia, and reductions in self-esteem and perceived social rank - Venting on social media negatively predicted positive affect and self-esteem and positively predicted paranoia and negative affect - Viewing social media newsfeeds predicted reductions in negative affect and paranoia - Perceptions of low social rank when using social media predicted low mood and self-esteem and high paranoia |
| Jakubowska A, Kaselionyte J, Priebe S, Giacco D. Internet use for social interaction by people with psychosis: A systematic review. Cyberpsychology, Behaviour, and Social Networking. 2019;22(5). | UK | To assess the most recent literature on how people with psychosis use online social networking and identify whether there has been any change in the findings of the previous review | Systematic review | - 13 included studies; 10 quantitative and all used surveys, 1 observational study of comments posted online, 2 qualitative studies - All studies published between 2013 and 2017 - All studies were in English | Total number of people with psychosis was 2662 | - 2 studies found that control groups and people with schizophrenia use the Internet similarly - Youth with psychosis were found to spend less time with online social networking compared to controls during acute illness - Facebook was found to be the most commonly accessed site, with Tumblr and YouTube being significantly less likely to be accessed by people with psychosis than mood disorder patients - Some evidence that people with SMI have similar use of social networking as general population in North America, but lower results were also reported in other studies - 1 study found that individuals with schizophrenia used the Internet more when symptom-free and found online activities helpful in managing their illness - Individuals using social networking are younger and had more time spent on it for education than nonusers - Young adults with psychosis were more likely to use Instagram and Snapchat compared with older people with schizophrenia - 6 studies looked at potential risk related to online social networking and found that there was no evidence that it led to worsening SMI - 3 studies found problems with harmful interactions between users with SMI and other people online: online comments are hurtful and permanent, there is concern about ability to verify identity of social contacts, patients were more likely to report feeling hurt online compared with control group, and people with psychosis largely ignore lack of anonymity online and associated risks of being identified as someone with SMI - Main identified purposes of online social networking were to establish new relationships, reconnect with others, and receive/give support - Some indication of social networking use to increase local community involvement |
| Buck B, Scherer E, Brian R, Wang R, Wang W, Campbell A, et al. Relationships between smartphone social behavior and relapse in schizophrenia: A preliminary report. Schizophrenia Research. 2019;208 (Behavioral Res):167-72. | USA | To evaluate whether smartphone-collected measures of social behaviour can serve as early behavioural indicators of relapse among individuals with schizophrenia. | Randomized controlled trial | - Average age of 37.11 - 36.07% white, 29.51% Black, 6.56% Pacific Islander, 1.64% Native, 1.64% Asian, 21.31% multiracial, 3.28% declined to report race - 27.87% Hispanic/Latino, 72.13% non-Hispanic/Latino - 9.84% some high school, 31.15% high school, 26.23% some college, 8.20% associate's, 19.67% bachelor's, 4.92% master's or above - 72.13% unemployed, 9.84% part-time work, 14.75% full-time work, 3.28% working less than part-time - 1.64% living in substance use treatment facility, 6.56% living in supported housing, 73.77% living with family, 18.03% living independently - Lifetime hospitalizations: 70.49% 1-5x, 16.39% 6-10x, 6.56% 11-15x, 1.64% 16-20x, 3.28% 20+x, 1.64% declined to answer | 61 | - 27 relapse events occurred for 20 participants during the study period - The number and duration of outgoing calls, as well as the total number of incoming and outgoing text messages, were significantly associated with relapse - Relapse was related to reductions in digital use between 6am and 12pm, 12pm and 6pm, and 12am and 6am. - Outgoing call duration between 12am-6pm and 12am-6am was associated with relapse |
| da Costa MP, Chevalier A, Farreny A, Cassidy M, Leverton M, Toner S, et al. How would patients with psychosis like to be in contact with a volunteer: Face-to-face or digitally? PLoS ONE. 2019;14(5):e0216929. | UK | To explore the views and interests of patients with psychosis about different formats of volunteering, face-to-face or digitally. | Quantitative non-experimental | - Average age of 42.6 - Average 15.6 years since receiving diagnosis - 71.5% male; 28.5% female - 23.2% White; 14.6% Black-Caribbean; 19.2% Black African; 7.9% Black Other; 12.6% Bangladeshi; 3.3% Indian; 5.3% Asian Pakistani; 1.3% Asian Chinese; 12.6% Other - 68.3% live alone; 12.4% live with parents; 6.2% live with partner; 3.4% live with children; 9.7% live with others - 6% paid employment; 0.7% sheltered employment; 84% unemployed; 3.3% in training/education; 3.3% retired; 2.7% other - All participants were followed by a CMHT and taking medication - Average monthly income of 699.69 pounds and all receive state benefits - 59.9% did not have children - 63.6% reported having a close friend; 53.6% saw a friend in the last week - 36% expressed not feeling lonely at all; 23.3% slightly; 20% moderately; 10.7% very; 10% extremely | 151 | - 57.6% had not heard about these volunteering programs previously - Significant association between interest in getting face-to-face volunteering input with loneliness and quality of life as significant predictors - Interest in getting digital volunteering input predicted by age and years since diagnosis - Majority preferred having a volunteer who had lived experience as a patient in mental health care - 13.2% did not use technology - Majority of participants preferred face-to-face weekly encounters (41.4%) - Preference for digital interactions was once per week (30.9%) - Preferred means of contact were text (46.4%) - Interest: 57.6% interested in getting face-to-face and 37.1% digital volunteering support - Large portion of participants felt that organization should be responsible for any costs associated with volunteering - Within those digitally interested, more aimed to make a friend than to do activities - Within those who were not digitally interested, more aimed to do more activities than make a friend - Majority wanted volunteers to be in contact with their mental health team (73.8% of face-to-face preferring and 65.5% of digital preferring) |
| Fernandez-Sotos P, Fernandez-Caballero A, Gonzalez P, Aparicio AI, Martinez-Gras I, Torio I, et al. Digital Technology for Internet Access by Patients With Early-Stage Schizophrenia in Spain: Multicenter Research Study. Journal of medical Internet research. 2019;21(4):e11824. | Spain | 1) To analyze how patients with early-stage schizophrenia use Internet and social networks compared with healthy participants matched by age and gender 2) To examine which devices are utilized to access Internet resources | Quantitative non-experimental | - Patients: all with schizophrenia; 5 years or less since first episode; average time of evolution of the disorder was 2.6 years; average age 28.1 years; 53% men, 47% women; 29% basic education, 50% medium, 21% high - Healthy participants: average 27.9; 57% men, 43% women; 22% basic education, 37% medium, 41% high | 180 | - 83% of patients used a smartphone daily; 56% used a computer daily; 20% smart TV; 10% game console; 9% tablet - Healthy participants used technology significantly more than patients except for game consoles - 11.1% of patients and 3.3% of healthy participants used Wikipedia - Age: Significantly higher use of smart TV in younger age groups in patients - Education: Significantly higher use of a computer with higher levels of education in patients - Rural vs. urban: Significantly higher use of game console and Smart TV in urban setting vs. rural in patients - 5.6% of patients did not indicate using any search engine, compared with 0% of healthy participants - Entertainment: healthy participants used smartphones, game consoles, and smart TVs significantly more for entertainment than patients - Work: healthy participants used computers significantly more than patients for work - Socialization: healthy participants used smartphones significantly more for socialization than patients - Shopping: healthy participants used computers and smartphones significantly more than patients for shopping |
| Spanakis P, Heron P, Walker L, Crosland S, Wadman R, Newbronner E, et al. Use of the Internet and Digital Devices Among People With Severe Mental Ill Health During the COVID-19 Pandemic Restrictions. Frontiers in Psychiatry. 2021;12:732735. | UK | 1) To identify the extent to which people with SMI have been using the Internet, whether socio-demographic and health characteristics had any influence on this, and whether Internet use was associated with changes in mental or physical health 2) To understand what people have been using the Internet for and what barriers exist to this | Quantitative non-experimental | - Average age of 50.5 - 51% men, 47.4% women, 1.6% transgender - 17.7% people from other than White ethnic background - 48.5% residing in high/very high deprivation areas - 51.2% diagnosed with psychosis - 33% completed survey online and over the phone, and 67% completed via the post - 16.6% decline in financial situation during pandemic - 37.9% primary care patients, 61% secondary care - 40.3% decline in mental health during pandemic - 32.2% decline in physical health during pandemic | 367 | - During pandemic, 37.1% were Internet users, while 61.6% were limited or non-users - Most participants owned a digital device and had access to the Internet from home - Around half rated knowledge of the Internet as good or higher and reported no knowledge gap - Of those reporting a knowledge gap, 59.3% were interested in learning more about the Internet - Those who reported a decline in their mental health since beginning of pandemic were almost twice as likely to have used the Internet a lot during the pandemic - More people with bipolar disorder (51.9%) reported a mental health decline compared to people with psychosis (34.8%) - Most common barriers were lack of interest in using Internet (28.3%), finding the Internet too difficult to use (27.9%), being concerned about personal security (24.3%), and concerns about privacy (22.1%) - Younger adults were 5-6x more likely to use the Internet a lot during the pandemic, compared to those 66+ - Participants with bipolar disorder were about 4x more likely to use the Internet a lot during the pandemic compared to those with psychotic disorder - Decline in mental health was significantly associated with use of the Internet - 88.9% used Internet for entertainment or information, 84.8% to stay in touch with friends or family, 84.3% online shopping |
| Chang KC, Chang YH, Yen CF, Chen JS, Chen PJ, Lin CY, et al. A longitudinal study of the effects of problematic smartphone use on social functioning among people with schizophrenia: Mediating roles for sleep quality and self-stigma. Journal of Behavioral Addictions. 2022;11(2):567-76. | Taiwan | 1) To test whether sleep quality mediated associations between problematic smartphone use and social functioning 2) To test whether self-stigma mediated associations between problematic smartphone use and social functioning | Quantitative non-experimental | - Average age 41.34 - 45.6% male - 12.14 average years of education - 36.8% employed - 32.1% had a physical disease - 8.8% lived alone | 193 | - Problematic smartphone use, sleep quality, and self-stigma were significant predictors for social functioning - Over time, participants demonstrated poorer social functioning and poorer sleep quality - Problematic smartphone use was a significant predictor for sleep quality and self-stigma |

**Appendix D**

**Summary of Assigned Categories for Each Included Study**

| **Author, Year, Country** | **Differences and Similarities in ICT Use Between Participants with Psychosis and Other Populations** | **Moderators of ICT Use and Access** | **Potential Benefits of ICT** | **Potential Risks of Harm of ICT** |
| --- | --- | --- | --- | --- |
| Haker, Lauber, & Rössler, 2005, Switzerland |  |  | **✓** |  |
| Beebe et al., 2010, USA | **✓** | **✓** | **✓** | **✓** |
| Spinzy et al., 2012, Israel |  | **✓** | **✓** |  |
| Grossman et al., 2020, USA & Canada |  | **✓** |  |  |
| Naslund et al., 2014, USA |  |  | **✓** | **✓** |
| Biagianti, Quraishi SH, Schlosser DA., 2018, USA |  |  | **✓** |  |
| Ben-Zeev et al., 2013, USA |  | **✓** | **✓** | **✓** |
| Schrank et al., 2010, Austria |  | **✓** | **✓** | **✓** |
| Sharma-Misra et al., 2023, South Africa |  | **✓** | **✓** |  |
| Tanriverdi Olug et al., 2024, Turkey |  | **✓** | **✓** |  |
| Miller at al., 2015, USA |  | **✓** | **✓** | **✓** |
| Thomas et al., 2017, Australia |  | **✓** | **✓** |  |
| Berry, Emsley, & Lobban, 2018, UK | **✓** | **✓** | **✓** | **✓** |
| Jakubowska et al., 2019, UK | **✓** |  | **✓** | **✓** |
| Buck et al., 2019, USA |  | **✓** |  |  |
| da Costa et al., 2019, UK | **✓** | **✓** | **✓** |  |
| Fernandez-Sotos et al., 2019, Spain | **✓** | **✓** |  |  |
| Spanakis et al., 2021, UK | **✓** | **✓** |  | **✓** |
| Chang et al., 2022, Taiwan |  | **✓** |  | **✓** |
